# Targeting populations at higher risk for malaria: a survey of national malaria elimination programmes in the Asia Pacific

**DOI:** 10.1186/s12936-016-1319-1

**Published:** 2016-05-10

**Authors:** Shawn Wen, Kelly E. Harvard, Cara Smith Gueye, Sara E. Canavati, Arna Chancellor, Be-Nazir Ahmed, John Leaburi, Dysoley Lek, Rinzin Namgay, Asik Surya, Garib D. Thakur, Maxine Anne Whittaker, Roly D. Gosling

**Affiliations:** Global Health Group, University of California, 550 16th St, 3rd Floor, San Francisco, CA 94158 USA; Centre for Biomedical Research, Burnet Institute, 85 Commercial Road, Melbourne, VIC Australia; Department of Clinical Tropical Medicine, Faculty of Tropical Medicine, Mahidol University, 420/6 Ratchawithi Road, Ratchathewi, Bangkok, 10400 Thailand; University of Queensland School of Public Health, Level 1, Public Health Building (887), Herston Rd, Herston, QLD 4006 Australia; Department of Microbiology, National Institute of Preventive and Social Medicine, Ministry of Health and Family Welfare, Mohakhali, Dhaka, Bangladesh; National Vector Borne Disease Control Programme, Ministry of Health and Medical Services, Honiara, Solomon Islands; The National Center For Parasitology, Entomology and Malaria Control, Ministry of Health, Corner street 92, Trapaing Svay village, Sankat Phnom Penh Thmey, Khan Sensok, Phnom Penh, Cambodia; Vector-borne Disease Control Programme, Ministry of Health, Gelephu, Royal Government of Bhutan; National Malaria Control Programme, Directorate General of Disease Control and Environment Health, Ministry of Health, Jakarta, Republic of Indonesia; Monitoring and Evaluation Division, Ministry of Health and Population, Kathmandu, Nepal; College of Public Health, Medical and Veterinary Sciences, Division of Tropical Health and Medicine, James Cook University, Townsville, QLD 4811 Australia

**Keywords:** Malaria elimination, High-risk populations, Mobile populations, Migrant populations, Hard-to-reach populations, Populations at higher risk, Asia Pacific

## Abstract

**Background:**

Significant progress has been made in reducing the malaria burden in the Asia Pacific region, which is aggressively pursuing a 2030 regional elimination goal. Moving from malaria control to elimination requires National Malaria Control Programmes (NMCPs) to target interventions at populations at higher risk, who are often not reached by health services, highly mobile and difficult to test, treat, and track with routine measures, and if undiagnosed, can maintain parasite reservoirs and contribute to ongoing transmission.

**Methods:**

A qualitative, free-text questionnaire was developed and disseminated among 17 of the 18 partner countries of the Asia Pacific Malaria Elimination Network (APMEN).

**Results:**

All 14 countries that responded to the survey identified key populations at higher risk of malaria in their respective countries. Thirteen countries engage in the dissemination of malaria-related Information, Education, and Communication (IEC) materials. Eight countries engage in diagnostic screening, including of mobile and migrant workers, military staff, and/or overseas workers. Ten countries reported distributing or recommending the use of long-lasting insecticide-treated nets (LLINs) among populations at higher risk with fewer countries engaging in other prevention measures such as indoor residual spraying (IRS) (two countries), spatial repellents (four countries), chemoprophylaxis (five countries), and mass drug administration (MDA) (three countries). Though not specifically tailored to populations at higher risk, 11 countries reported using mass blood surveys as a surveillance tool and ten countries map case data. Most NMCPs lack a monitoring and evaluation structure.

**Conclusion:**

Countries in the Asia Pacific have identified populations at higher risk and targeted interventions to these groups but there is limited information on the effectiveness of these interventions. Platforms like APMEN offer the opportunity for the sharing of protocols and lessons learned related to finding, targeting and successfully clearing malaria from populations at higher risk. The sharing of programme data across borders may further strengthen national and regional efforts to eliminate malaria. This exchange of real-life experience is invaluable to NMCPs when scarce scientific evidence on the topic exists to aid decision-making and can further support NMCPs to develop strategies that will deliver a malaria-free Asia Pacific by 2030.

## Background

Significant progress has been made in reducing malaria morbidity and mortality worldwide. Between 2000 and 2014, the global malaria mortality rate fell by 60 %, attributable to a combination of more developed economies, increased funding, improved surveillance and case-management, and scale-up of interventions [1–3]. In the Asia Pacific region, malaria deaths have declined by 86 %, with many countries in this region demonstrating exceptional individual progress [3]. For example, the Philippines reduced its number of malaria cases by 86 % between 2000 and 2014, with one-third of provinces having eliminated malaria as of 2013 [3, 4]. In Bhutan, confirmed cases declined 99 % from 2000 to 2014, with only 19 locally transmitted cases in 2014 [3]. Sri Lanka has reported zero locally transmitted malaria cases since 2012 [3].

With such impressive gains, many countries have set or are currently setting national or subnational malaria elimination goals. At the same time, global and regional strategic support for these efforts is growing, evidenced by the World Health Organization’s (WHO) new Global Technical Strategy for Malaria 2016–2030 [5] and Strategy for Malaria Elimination in the Greater Mekong Sub-region 2015–2030 [6], as well as the newly defined Asia Pacific regional elimination goal of 2030 [7, 8]. Despite the global and regional momentum towards elimination, epidemiological and operational challenges remain. Regional collaborations can address some of these challenges including cross-border importation risk and reaching populations that are often migrant, mobile, stigmatized, or hard to reach [2, 5, 9, 10]. The Asia Pacific Malaria Elimination Network (APMEN) has demonstrated leadership in this domain, playing a critical role in sharing best practices and fostering cross-border collaboration among its 18 country partners in the Asia Pacific [11].

Appropriately targeting populations at higher risk for malaria is crucial as it helps ensure that surveillance and response activities reach all populations at risk of malaria; thereby shrinking parasite reservoirs and interrupting transmission in elimination settings [12]. To date, most research conducted has focused upon defining at-risk populations and the factors that put them at risk of malaria infection. The current understanding is that populations at higher risk for malaria are clustered in discrete geographical areas and in subpopulations with certain demographic, social, and behavioural risk characteristics [2, 9, 13–15]. These population groups maintain higher malaria transmission rates than the surrounding population, which poses both a health threat to these populations and a risk of onward malaria transmission in an eliminating country [13].

In the Asia Pacific region, populations at higher risk are predominantly adult males who are exposed to infectious mosquitoes in their work as rubber tappers [16–18], forest workers [19–21], miners [4, 21, 22], military personnel [22–25], or farmers practicing forms of shifting agriculture [26]. Populations along national borders, such as the Laos–Cambodia, Vietnam–Cambodia, and Thailand–Myanmar borders [27], are also considered at higher risk, as many border regions near forest and forest fringe areas are highly receptive to malaria, have limited access to health facilities and prevention programmes, have a higher degree of population mobility, and may be affected by political or ethnic conflicts [1, 28]. Other identified populations at higher risk in the Asia Pacific region include laborers returning from malaria-endemic regions in other countries [29–32] or within the same country [23, 33] who travel to non-endemic regions, mobile ethnic groups [18, 22, 24, 31], rural indigenous communities [4, 22, 23], migrants [24, 34, 35], and persons displaced due to civil unrest [36]. These populations not only have limited access to prevention, diagnostic testing, and treatment services, but their undiagnosed and untreated parasitaemia may also contribute to transmission to surrounding populations in receptive areas [10, 37–39].

Despite efforts to characterize populations at higher risk in the Asia Pacific, the existing research does not specify how National Malaria Control Programmes (NMCPs) characterize populations considered to be at higher risk. There is also limited knowledge of the range and effectiveness of strategies and interventions implemented by NMCPs targeting populations at higher risk. At the APMEN Surveillance and Response Working Group meeting in Wuxi, China in September 2013, leaders of NMCPs described the need to identify populations at higher risk in order to develop appropriate strategies for targeting surveillance, prevention, and treatment activities. Countries also noted the need to share strategies and interventions across countries to learn about best practices. In response, APMEN developed a qualitative, free-text based survey on populations at higher risk, which was disseminated amongst the NMCPs of the APMEN partner countries. This survey sought to identify and investigate (1) populations at higher risk for malaria as characterized by NMCPs in the Asia Pacific; (2) actions targeting populations at higher risk with respect to health information, malaria control, prevention, treatment, monitoring and evaluation, and surveillance; and (3) common challenges faced by NMCPs in designing, implementing and assessing impact of interventions targeted to populations at higher risk as well as any self-identified gaps in technical capacity and tools. By leveraging the APMEN network, country-specific information on population groups at higher risk of malaria can be gathered and used to guide future strategic decision-making and engagement on this important issue by these country programmes. Effectively targeting populations at higher risk is necessary to achieve the regional elimination goal of a malaria-free Asia Pacific by 2030, and to ensure equity in malaria diagnosis, treatment and prevention services across all population groups.

## Methods

The survey tool was developed and piloted and then revised. Survey questions pertained to: populations at higher risk defined in country-specific malaria programmes; outreach activities providing malaria health information (i.e. dissemination of Information, Education, and Communication (IEC) materials, channels of delivery, and key messages); prevention, diagnosis, and treatment interventions within and between countries (i.e. vector control, screening, chemoprophylaxis, mass drug administration (MDA), and personal protection); surveillance and tracking (i.e. availability of geo-referenced or spatial data, mass blood surveys); monitoring and evaluation (M&E); and self-identified challenges and needs.

In September 2014 the survey tool was distributed to programme managers of NMCPs of 17 APMEN partner countries: Bangladesh, Bhutan, Cambodia, China, Democratic People’s Republic of Korea, India, Indonesia, Lao People’s Democratic Republic, Malaysia, Nepal, Philippines, Republic of Korea, Solomon Islands, Sri Lanka, Thailand, Vanuatu, and Vietnam (since then Papua New Guinea has joined APMEN, making 18 country partners).

Survey responses were entered and analysed in Microsoft Access 2010. Queries were developed across each survey theme and the responses were analysed manually to account for differences in terms and definitions used across respondents. Categories were developed from responses and tabulated.

## Ethical considerations

The survey was certified as exempt from ethical review by the University of California, San Francisco Committee on Human Research.

## Results

Of the 17 APMEN country partner NMCPs invited to participate in the populations at higher risk survey, 14 country programmes responded by July 2015. Four APMEN country partners (India, Lao People’s Democratic Republic, Sri Lanka, and Papua New Guinea) are not represented in the following survey results. In total 16 surveys were received (Nepal and Vietnam each submitted two surveys). Seven respondents identified as the manager or director of the NMCP; six respondents identified as NMCP staff; two surveys were returned without identifying information; one survey respondent identified as working closely with and in an advisory capacity to the NMCP.

### Identification and characterization of populations at higher risk

NMCP staff identified and described a wide set of populations at higher risk with characteristics, behaviours, and risk factors that fall into three main categories.

#### Work environments that place populations in malaria-receptive areas

Eleven of the 14 respondents identified work environments that place populations in high-transmission or receptive areas (Table 1). Receptivity is described as “the abundant presence of anopheline vectors and the existence of other ecological and climatic factors favouring malaria transmission” [40]. One of the most common work environments that place people at higher risk for malaria were forests and forest-fringe areas (reported by seven countries), including wood cutters, rattan collectors, charcoal makers, loggers, and forest hunters. Forest workers often live where they work, sleeping in poorly protected make-shift housing structures or completely unprotected outdoors. Agricultural workers (reported by seven countries), ranging from plantation workers to those practicing shifting agriculture, were also identified as being at higher risk. Military personnel were reported by five counties. Other groups cited include workers in development project and construction sites in the Philippines and Democratic People’s Republic of Korea, students attending school in malaria-receptive areas in Bhutan, miners in Indonesia and the Philippines, and research surveyors in Malaysia.

**Table 1:** Characterization of populations at higher risk for malaria as identified by APMEN country partners

	BGD	BTN	KHM	CHN	PRK	IDN	MYS	NPL	PHL	KOR	SLB	THA	VUT	VNM
Work														
Forest workers	•		•			•	•	•	•					•
Agricultural workers	•	•			•	•	•			•				•
Military		•	•				•		•	•				
Development project and construction site workers					•				•					
Miners						•			•					
Students		•												
Research surveyors							•							
Other (i.e. monks, civil/government employees)		•									•			
Mobility and Migration														
Migrant workers		•	•	•			•	•	•					•
Mobile populations at country borders			•	•				•						•
Local and foreign tourists									•					
Indigenous nomads													•	
Other (i.e. nondescript mobile and migrant populations)											•		•	
Demographics														
Ethnic minorities, indigenous people							•		•				•	•
Refugees, internally displaced populations								•	•					
Low socioeconomic status poor/homeless								•			•		•	
Geographically remote/isolated	•										•		•	•
Time spent in malaria endemic regions			•							•		•		
Pregnant women, infants and children						•								

The categories are not mutually exclusive

Country abbreviations (adopted from the United Nations country abbreviations). *BGD* Bangladesh, *BTN* Bhutan, *KHM* Cambodia, *CHN* China, *PRK* Democratic People’s Republic of Korea, *IDN* Indonesia, *MYS* Malaysia, *NPL* Nepal, *PHL* Philippines, *KOR* Republic of Korea, *SLB* Solomon Islands, *THA* Thailand, *VUT* Vanuatu, *VNM* Vietnam

#### Mobility or movement between malaria-endemic and non-endemic areas

Nine out of 14 respondents identified populations whose mobility or movement increases risk of contracting malaria (Table 1). Migrant workers are highly mobile and were considered by seven countries as populations at higher risk. Chinese overseas workers returning from malarious regions in Africa and Southeast Asia, as well as workers returning to Nepal from India, were identified as serious importation risks as they move from endemic to non-endemic areas between countries. Mobile populations, especially at the borders between China and Myanmar, Nepal and India, and Lao Peoples’ Democratic Republic, Cambodia, and Thailand, were also noted as having higher risk for malaria. Other groups who are mobile for reasons not directly attributable to informal or formal work opportunities include local and foreign tourists in the Philippines and indigenous nomads in Vanuatu.

#### Demographic factors

Eleven APMEN country partners included demographic factors in their descriptions of populations at higher risk, such as low socioeconomic status, internally displaced populations and refugees, the urban homeless and extreme poor, ethnic minorities, geographically isolated and remote populations, and those living near the Demilitarized Zone in the Republic of Korea (Table 1). Risk groups were not disaggregated by gender or age, except for Indonesia, which reported pregnant women, infants and children as groups at higher risk. Other influencing factors considered by NMCPs to increase risk, such as political and social unrest, armed conflict, and natural disasters, are captured in Table 1.

### Interventions targeting populations at higher risk

APMEN country partners identified a range of malaria prevention, diagnosis and treatment interventions targeting populations at higher risk.

#### Dissemination of information, education, and communication materials

Thirteen of the 14 respondents reported disseminating malaria-related IEC materials, making it the most commonly applied intervention utilized by APMEN country partners to target populations at higher risk (Table 2). Seven countries use the same IEC strategy across all populations at higher risk. Six countries use a mix of strategies depending on the intended audience; these countries reported using a combination of messages, channels and/or delivery mechanisms to reach different population groups. One country (China) did not report using this strategy in targeting populations at higher risk. Most countries use printed materials (reported by nine countries) or mass media such as television and radio (reported by six countries) to disseminate health information. Seven countries reported leveraging community action groups or campaign days, including long-lasting insecticide-treated net (LLIN) distribution days, for health information delivery. Schools (reported by three countries) and billboards (reported by two countries) were also reported as channels for disseminating malaria information.

**Table 2:** APMEN country partner strategies for disseminating malaria health information to populations at higher risk

	BGD	BTN	KHM	CHN	PRK	IDN	MYS	NPL	PHL	KOR	SLB	THA	VUT	VNM
Strategies deployed across different risk groups														
Same strategy for all	•				•	•	•	•			•			•
Mix of strategies		•	•						•	•		•	•	
Key messages														
Prevention and personal protection (e.g. net usage, treated clothing)		•	•		•	•	•	•	•	•	•	•	•	•
Malaria signs and symptoms	•	•	•		•	•	•	•	•		•		•	
Encouraging treatment-seeking	•	•	•		•		•	•	•		•	•	•	•
Encouraging prompt diagnosis					•	•		•	•		•	•	•	•
Compliance with treatment					•		•				•	•		
Channels														
Printed materials (i.e. leaflets, pamphlets)	•	•			•		•	•		•	•	•		•
Mass media (i.e. broadcast, news, TV, radio)		•	•		•						•	•		•
Campaign days (i.e. World Malaria Day, LLIN distributions, health surveys)		•						•	•				•	
Community Action Group		•				•			•					
Billboards		•						•						
Schools		•						•					•	
Delivered by														
Community health workers	•		•		•	•	•	•	•	•	•	•	•	•
Malaria health volunteers	•				•		•	•	•			•	•	
Employer (i.e. rubber company, military, construction project company)		•	•				•		•	•				•
Local representatives/authorities	⊙												•	•
School teachers		•										•	•	
Epidemiology/entomology research teams	•													
Social organizations		•												•

⊙ Small-scale pilot or in planning stages only

*BGD* Bangladesh, *BTN* Bhutan, *KHM* Cambodia, *CHN* China, *PRK* Democratic People’s Republic of Korea, *IDN* Indonesia, *MYS* Malaysia, *NPL* Nepal, *PHL* Philippines, *KOR* Republic of Korea, *SLB* Solomon Islands, *THA* Thailand, *VUT* Vanuatu, *VNM* Vietnam

IEC messaging centered on malaria prevention and personal protection methods, including the proper use of insecticide-treated nets (ITNs) (reported by 12 countries). Other key messages include malaria signs and symptoms (reported by 10 countries), promotion of prompt diagnosis and early treatment (reported by eight and 11 countries, respectively), and information on proper compliance with treatment (reported by four countries). These messages are mainly delivered by community health workers (CHWs) (reported by 12 countries). Other important actors include malaria health volunteers (reported by seven countries), employers such as the military or companies overseeing large construction or mining projects (reported by six countries), local authorities (reported by three countries), school teachers (reported by three countries), researchers in the field conducting epidemiological and entomological studies (reported by one country), and social organizations (reported by two countries).

#### Diagnostic screening and treatment

Eight respondents reported conducting diagnostic screening or testing among populations at higher risk of malaria (Table 3). Most screening activities focus upon mobile populations arriving from malaria endemic areas in other countries or moving between endemic and non-endemic areas within the same country. Two countries (Bhutan, Malaysia) screen incoming migrant workers at country borders; Nepal is planning border post screening with Global Fund grant funding. Other populations screened are military personnel (Malaysia, Nepal), returning overseas workers (China), returning overseas United Nations peacekeepers (Bangladesh, Nepal), and incoming foreign students (Malaysia). Screening migrants moving between non-endemic and endemic parts within the country’s borders is conducted by two countries (Indonesia and in a small pilot, Vanuatu). Periodic screening is conducted at logging camps, plantations, and native settlements in malarious areas in Indonesia, Malaysia, and the Philippines. The Philippines reported the planned establishment of a malaria elimination hub in pre-elimination and elimination provinces to improve capacity for prevention, diagnosis, case management, and treatment.

**Table 3:** Targeted interventions for screening, treatment, and prevention of malaria in populations at higher risk among APMEN country partners

		BGD	BTN	KHM	CHN	PRK	IDN	MYS	NPL	PHL	KOR	SLB	THA	VUT	VNM
Diagnostic screening or testing															
Of overseas UN peacekeepers upon return		•							•						
Of military								•	•						
Of incoming migrant workers at borders			•					•	⊙						
Of incoming foreign students, volunteers								•							
Of returning overseas workers					•										
Of individuals moving between endemic and non-endemic regions within the same country							•							⊙	
Of workers at logging camps, plantations, native settlements in malarious area							•	•		•					
Treatment															
Available at health facility			•						•				•	•	•
Prevention															
Personal protection and vector control											•				
Long-lasting insecticide treated net		•	•			•	•		•	•		•	•	•	•
Indoor residual spraying			•							•					
Spatial repellents			•						•			•			•
Chemoprophylaxis															
For military								•							
For pregnant women														•	
For travelers									•	•	•				
For individuals moving between endemic and non-endemic regions									⊙						
Mass drug administration															
Implemented						•								■	■
Planned or piloted		⊙		⊙											

**■** Conditional MDA (e.g. VNM only applies MDA with ACTs in case of outbreaks), ⊙ Small-scale pilot or in planning stages only

*BGD* Bangladesh, *BTN* Bhutan, *KHM* Cambodia, *CHN* China, *PRK* Democratic People’s Republic of Korea, *IDN* Indonesia, *MYS* Malaysia, *NPL* Nepal, *PHL* Philippines, *KOR* Republic of Korea, *SLB* Solomon Islands, *THA* Thailand, *VUT* Vanuatu, *VNM* Vietnam

Throughout the survey, some country respondents referenced passive approaches to prevention, diagnosis and treatment. Five countries reported that anti-malarial treatments are available at public and/or private health facilities. Although the survey did not inquire about passive case detection and treatment specifically, this was a recurring theme in the survey responses.

#### Prevention

Thirteen of the 14 respondents employ prevention interventions targeted to populations at higher risk (Table 3). The interventions fall into the categories of personal protection and vector control, chemoprophylaxis, and MDA.

Eleven of the respondents reported targeting these populations with vector control and personal protection methods. Ten countries distribute or recommend the use of LLINs to targeted risk groups, which included military personnel and peacekeeping forces deployed to malarious regions. The Solomon Islands advises travellers to use insecticide-treated nets and repellents, but does not provide those tools. Hotels in endemic areas in Bhutan are mandated to provide LLINs or spatial repellents to guests. Health facilities in Thailand, Vietnam, and Bhutan provide bed nets passively, requiring populations at higher risk to come to the facility of their own accord. Only Bhutan and the Philippines reported targeting specific risk groups with indoor residual spraying (IRS).

Five NMCPs reported that they administer, or recommend the use of, anti-malarials as chemoprophylaxis to at least one risk group. These drugs suppress the blood stage of the *Plasmodium* parasite and prevent clinical malaria. Malaysia and Vanuatu administer chemoprophylactic drugs to military personnel and pregnant women, respectively. Nepal advises people leaving the country and travelling to malaria-endemic countries in Africa to use chemoprophylaxis while travelling. The Republic of Korea and the Philippines provide similar recommendations for overseas travelers. Nepal plans to include chemoprophylaxis for individuals moving between endemic and non-endemic regions in the country’s elimination guidelines once it enters the prevention of reintroduction phase (POR).

The Democratic People’s Republic of Korea is the only APMEN country partner that has regularly implemented MDA; after successfully piloting mass primaquine preventive treatment in 2002 with WHO technical support, the country reports using mass primaquine preventive treatment seasonally in high burden provinces. Other countries reported carrying out MDA but only in certain situations. For example, Vietnam reported using MDA with artemisinin-based combination therapy (ACT) in cases of malaria outbreaks. Cambodia is piloting targeted malaria elimination, which includes MDA with ACT and low dose primaquine. At the time of the survey, Bangladesh was drafting a protocol to pilot targeted malaria elimination in a few endemic regions; however, it has not yet begun due to logistical reasons.

### Surveillance activities targeting populations at higher risk

Information on two specific surveillance activities was captured in the survey: mass blood surveys and the collection of geo-referenced or spatial malaria case data. Specifically, the survey inquired if malaria programmes used these activities to collect information on population groups considered at higher risk of malaria. For the most part, while a majority of the countries engaged in these activities on some level (13 of 14 countries use mass blood surveys and/or geo-referenced data), many did not report tailoring these activities to populations at higher risk but instead based these activities on geographical or situational factors, such as in high-transmission zones or for outbreak response.

#### Mass blood surveys

Eleven of the 14 country respondents implement mass blood surveys in some capacity to screen populations at higher risk (Table 4). Among those, five countries reported using mass blood surveys in reactive case detection (RACD) activities. An additional country (Nepal) is planning a pilot of mass blood surveys for the surrounding 50 households of an index case with the aim of identifying asymptomatic cases within the community. Two countries (Malaysia, Thailand) reported using mass blood surveys during proactive case detection. Additionally, the Philippines, which reported previously completing prevalence surveys among indigenous populations using mass blood surveys, is planning to use rapid diagnostic tests (RDTs) to screen other populations at higher risk in the country. Two countries (China, Vietnam) use mass blood surveys in conjunction with outbreak response and another two countries (Bhutan, Indonesia) use it in high transmission zones. Bhutan uses mass screening, including mass blood surveys, in major project development sites with approval from project authorities. The Republic of Korea uses serology to monitor those living in malaria endemic regions as well as the armed forces, the two most at-risk groups in the country.

**Table 4:** Surveillance activities among APMEN country partners

	BGD	BTN	KHM	CHN	PRK	IDN	MYS	NPL	PHL	KOR	SLB	THA	VUT	VNM
Mass blood surveys														
In project development sites		•												
In high transmission zones		•				•								
Prevalence surveys			•						•					
Focal screen and treat (reactive case detection)				•	•		•	⊙				•		•
Focal screen and treat (proactive case detection)							•		⊙			•		
Outbreak response				•										•
Method: serology										•				
Geo-Referenced and spatial data														
Mapping with no geo-referencing		•	•			•	•	•		•				•
SMS based reporting								⊙						
Geo-referenced system		⊙					⊙	⊙			■	•	■	⊙

⊙ Small-scale pilot or in planning stages only, **■** In pre-elimination provinces only

*BGD* Bangladesh, *BTN* Bhutan, *KHM* Cambodia, *CHN* China, *PRK* Democratic People’s Republic of Korea, *IDN* Indonesia, *MYS* Malaysia, *NPL* Nepal, *PHL* Philippines, *KOR* Republic of Korea, *SLB* Solomon Islands, *THA* Thailand, *VUT* Vanuatu, *VNM* Vietnam

#### Geo-referenced and spatial data

Ten of the 14 countries map their case data (Table 4). Most countries described their mapping activities as based on geography and not on population groups. Only Malaysia reported monitoring populations at higher risk in their mapping activities. Of the ten countries that report mapping their case data, seven countries (Bhutan, Cambodia, Indonesia, Malaysia, Nepal, South Korea, Vietnam) conduct mapping but do not currently use geo-referenced data on a large scale. Most of the countries aggregate case data at specified intervals and transfer the data to maps using geographic information system (GIS) software. The survey did not inquire as to the resolution level of mapping activities. However, of the six countries that included this information in their responses, five countries noted that mapping takes place at the village level. In pre-elimination provinces in Vanuatu mapping takes place at the household level.

In Malaysia, malaria is a notifiable disease and all cases are entered into a centralized computer system; Malaysia reported that their current surveillance system is able to monitor inter-regional migration of cases as well as the incidence of malaria among groups at higher risk. Thailand is the only country that reported the consistent use of real time, geo-referenced data. Four countries (Bhutan, Malaysia, Nepal, Vietnam) plan to use or are piloting real-time, geo-referenced data. After a successful pilot programme in one district of Bhutan, the country is planning to expand the use of geo-referenced data with mobile technology. Malaysia is developing a new, web-based management system to collect, aggregate and map geo-referenced case data in real time. Nepal is planning to target populations at higher risk when it rolls out a new short message service (SMS) based reporting system and incorporates spatial analysis of that data into the malaria surveillance system. The Solomon Islands and Vanuatu reported using geo-referenced data in their reporting systems but this is only implemented in pre-elimination provinces.

### Monitoring and evaluation of interventions for populations at higher risk

The majority of APMEN country partner respondents reported not having robust M&E procedures for interventions targeting populations at higher risk. Four of 14 countries (Philippines, Thailand, Vanuatu, and Vietnam) reported that comparison of annual national or subnational level indicators, such as morbidity, mortality, ITN or IRS coverage, has been used to measure impact of interventions. The remaining ten country respondents did not report any operationalized plan for monitoring and evaluating interventions targeted to populations at higher risk. Bhutan, Nepal and Vanuatu reported that mobility and porous borders present challenges to M&E efforts.

### Challenges to targeting populations at higher risk

The most common challenge (eight of 14 countries) reported by NMCPs to effectively target populations at higher risk for malaria elimination was lack of knowledge, surveillance tools, and capacity. Four programmes (Bhutan, Cambodia, China, Thailand) reported the need to understand current movement patterns of mobile and migrant populations. Three programmes (Nepal, Vanuatu, Vietnam) stated the need for GIS software and training, while one country (Vietnam) needs tools to implement active surveillance. Health information system strengthening was noted by one country (Solomon Islands) as a priority gap, and one country (China) noted the need for genotyping to differentiate between local and imported cases. Screening kits for malaria diagnosis using loop-mediated isothermal amplification (LAMP) and polymerase chain reaction (PCR), as well as diagnostic tools for G6PD deficiency, were identified by two countries (Bhutan, Solomon Islands) as priority issues in order to facilitate early and proper treatment. A need for technical assistance and implementation guidelines on methods for targeting populations at higher risk was reported by four countries (Bangladesh, Bhutan, Nepal, Vanuatu). Other needs identified by the countries included financial assistance (Vanuatu, Vietnam), vector control methods for exophagic vectors (Thailand), and regional data and information sharing (Malaysia). Four countries did not report on gaps or needs in this section of the survey.

## Discussion

Populations at higher risk for malaria who do not have access to prevention, diagnosis and treatment pose a threat to the Asia Pacific regional goal of malaria elimination by 2030 [2, 5, 10]. This survey of APMEN country partners showed that NMCPs in the Asia Pacific region have identified populations that are considered to be at higher risk of malaria infection in their respective countries. Some, but not all, countries have developed targeted interventions specifically for these population groups. Strategies to improve targeting surveillance, diagnosis and treatment, prevention and other malaria control interventions towards these population groups are needed.

### Who are the populations at higher risk targeted by NMCPs in the Asia Pacific?

All survey respondents identified the population groups considered at higher risk of malaria and provided detailed descriptions of these groups. Populations at higher risk fell into three general categories: work environments that place them in malaria-receptive areas, mobility or movement, and demographic factors, such as low socioeconomic status. It is important to recognize that these risk categories are not rigid; the example of indigenous groups of farmers practicing shifting agriculture in Bangladesh who move from non-malarious areas to malarious areas a few months every year highlights how demographics, work, and mobility interact to increase risk of malaria [26]. These categories are often broad, change over time and intersect considerably with one another, making targeting difficult. Further, as risk of transmission increases, these factors also create obstacles to accessing effective malaria prevention, diagnosis, and treatment services [41].

In order to target resources effectively towards groups that are actually at higher risk for malaria requires NMCPs to assess risk factors in greater detail. Tools are under development to assist NMCPs take a more standardized epidemiological approach to identifying populations at higher risk for malaria. The human population movement framework developed in Cambodia is an example of an integrated approach to assessing risk of malaria among a dynamic and interrelated set of categories [41]. Other methods for identifying higher risk groups include respondent-driven sampling [42, 43], case–control studies [44], social networking [45], venue-based studies [46, 47], health services mapping, applied anthropological studies [48–50], and multiple cross-sectional mixed methods studies [51]. In some cases, combinations of these methods may be most effective at identifying populations at higher risk of malaria.

The survey results reinforced the idea that many NMCPs in the Asia Pacific perceive similar population groups as being at higher risk for malaria. These similarities further underscore the importance of information sharing on methods and strategies to identify populations at higher risk of malaria among NMCPs in the Asia Pacific region. Additionally, sharing programme data on epidemiology, risk factors, outbreaks, and intervention coverage across borders may aid NMCPs to plan activities more efficiently.

### What interventions are used to target for populations at higher risk in the Asia Pacific?

The study revealed a few intervention areas for which the majority of NMCPs have developed and tailored approaches for populations at higher risk. Most respondents develop IEC materials and disseminate messaging using mass media or printed materials. Nearly half of those respondents use a mix of IEC strategies to target different population groups. IEC and behaviour change communications (BCC) strategies can positively impact malaria prevention and treatment-seeking behaviours, especially when rooted in community engagement [52]. For example, in Cambodia, a project based on positive deviance, a community-based approach to behaviour change, was found to be effective at increasing malaria knowledge and improving health-seeking behaviour among both the local population and mobile and migrant workers in the catchment area [53]. While IEC and BCC have been found to remain an effective intervention in low transmission settings there is evidence that the penetration of messaging can be uneven across population groups [43, 54, 55]. Given the widespread use of IEC materials to reach populations at higher risk among APMEN country partners, it is important that messaging, format, and delivery mechanisms are well-suited for the intended audiences. Utilizing a mixed-methods approach, as some NMCPs reported doing, will likely yield better results [52]. For example, some NMCPs work through employers or local authorities for the delivery of malaria messages. The Roll Back Malaria Partnership’s (RBM) Action and Investment to Defeat Malaria 2016-2030 [56] promotes a multisectoral approach to malaria elimination, including strengthening collaborations with private industries to reach at-risk populations with health messaging and other health services.

None of the NMCPs reported on systematic evaluations of the effectiveness of their IEC interventions among populations at higher risk. Indictors developed for the RBM Malaria Behavior Change Communication Indicator Reference Guide [57] may be adopted for local use to measure knowledge, attitudes, and practices among populations at higher risk. As transmission dynamics change continued operational research on how to best reach populations at higher risk with malaria messaging will be required.

The majority of NMCPs target populations at higher risk with preventative interventions, mainly LLINs. However, there are increasing reports of vector resistance to pyrethroids, the class of insecticides recommended for treating bed nets, throughout the Asia Pacific [3]. Vectors can exhibit physiological and behavioural resistance that undermine the efficacy of LLINs and contribute to residual transmission [58–61]. In some areas, including parts of the Greater Mekong Subregion (GMS), residual transmission is hypothesized to be a result of heterogeneous human activity coupled with diverse vectors that tends to bite early and outdoors [60, 62]. Despite these challenges, LLINs are still considered a critical vector control tool but additional personal protection interventions are needed [60]. According to the survey results only a minority of countries target populations at higher risk for malaria with IRS, spatial repellents, chemoprophylaxis, or MDA, indicating that these approaches may be under-utilized. To address the challenges of insecticide resistance, countries in the Asia Pacific should prioritize improving coverage of populations at higher risk with existing vector control interventions while also field testing new, supplementary tools [63, 64].

Fewer countries target populations at higher risk for diagnostic screening and treatment activities than preventative activities. The emergence and spread of artemisinin and multidrug resistance in the GMS [27] called for an emergency response with a focus on reaching high-risk and hard-to-reach populations with full coverage of core malaria interventions in priority areas [65]. Mathematical modelling suggests that the elimination of artemisinin-resistant malaria necessitates high coverage of ACT in addition to LLINs [66]. Targeting populations at higher risk for confirmed diagnosis and quality treatment is imperative to achieve elimination. Test, treat and track activities for populations at higher risk should be scaled up in Asia Pacific countries to contain artemisinin resistance and expand universal coverage of core malaria interventions.

Surveillance is a critical intervention for malaria elimination. Active malaria surveillance approaches include reactive and proactive activities, both of which are used by NMCPs in the Asia Pacific in combination with mass blood surveys. These surveillance activities can potentially increase access to diagnosis and treatment of malaria among populations at higher risk or in areas of suspected transmission [2, 9, 12]. However, in low transmission settings with a high proportion of subpatent infections, active case detection will likely miss infections [2, 13]. New sensitive diagnostic tools appropriate for field use are required to increase the effectiveness of identifying a greater number of infections during active case detection and improve rapid and accurate diagnosis and treatment [67]. Some countries reported the desire to utilize sensitive diagnostic tools such as LAMP and PCR to capture low density infections; this may represent an opportunity to expand operational research on these diagnostic tools in the Asia Pacific region among populations at higher risk.

Despite some of the active surveillance activities in place to reach populations at higher risk, passive approaches to prevention, diagnosis and treatment are still common. Five countries noted that treatment was available at health facilities and three countries reported that LLINs can be obtained at these facilities. These interventions are dependent upon populations at higher risk seeking out and effectively accessing care. However, the factors that place populations at higher risk—lower socioeconomic status, mobility, displacement, or living in geographically remote areas—limit access to and use of health facilities [2]. A reliance on passive case detection will miss asymptomatic infections, especially among hard-to-reach populations, thereby hindering equitable access to prevention, diagnosis and treatment among populations at higher risk for malaria [68–70]. The use of community and village health workers is a step towards increasing this access to vulnerable groups [5, 9]. CHWs or peer-group educators that move or work with vulnerable populations, similar to those used in HIV control, may further improve penetration of preventive health messages and reduce malaria among higher risk groups [71].

### How can targeting populations at higher risk be improved in the Asia Pacific?

Human population movement was reported as a serious obstacle in reaching populations at higher risk with effective malaria interventions. Human population movement poses the risk of malaria importation into low endemic or malaria-free areas and also the potential for the spread of antimalarial drug resistance [10, 48]. Some respondents have developed an approach to mitigate this importation risk by planning or conducting surveillance activities targeting incoming migrants in border areas, such as in Cambodia [10]. Several countries noted the need to better understand human population movement throughout the region to better target mobile and migrant populations.

Respondents identified a need for technical support on surveillance. Activities such as conducting blood surveys in hard-to-reach locations or incorporating spatial data into national surveillance systems are resource and time intensive. However, there is a demonstrated interest among NMCPs in improving surveillance systems. Four countries are currently piloting or planning to incorporate SMS-based or geo-referenced data into their surveillance systems. There is an opportunity to better integrate populations at higher risk within these new or updated surveillance systems. An increase in the amount and quality of surveillance data will allow for patterns and trends in populations at higher risk to emerge. This information is essential for identifying the best interventions and methods to target populations at higher risk.

The majority of respondents do not conduct M&E to evaluate the impact of interventions in reducing transmission among populations at higher risk. The survey was aimed at respondents that could best provide information on at-risk populations; depending on the malaria programme this may not be the same person that is able to speak extensively on specific M&E activities. However, of the four countries that did report on M&E efforts, annual national level indicators, such as morbidity, mortality, ITN or IRS coverage, are used to measure the success of targeted interventions. These general indicators are broad in scope and scale. Evidence-based decisions on how to use programme resources are not possible without a reliable way to assess if interventions reach those who they are intended to reach and if they effectively reduce transmission. Well-adapted M&E efforts provide valuable information on which populations should be prioritized with which interventions and the effectiveness of interventions used to serve them. Similarly, while NMCPs identified population groups at higher risk, it is possible there are subpopulations that have been unintentionally overlooked as a result of non-specific M&E efforts—in the absence of robust M&E systems, the combination of interventions necessary to effectively target populations at higher risk will remain unknown.

To achieve and sustain malaria elimination, countries must provide universal access to malaria prevention, diagnosis, and treatment. This can be achieved by proactively tracking populations at higher risk, targeting with evidence-based interventions whilst respecting individual rights, and evaluating intervention impact. It is important for APMEN country partners, who are aiming to eliminate malaria from the region by 2030, to continue to engage in information-sharing and improving the evidence base on populations at higher risk in the Asia Pacific.

## Limitations

The results in this report are from the survey responses received from national malaria programme staff. The survey was comprised of open-ended, free text qualitative questions. Not all respondents provided answers to every question and due to time constraints there was no individual follow up. As a consequence, responses are not standardized and there will likely be gaps in the information presented. Additionally, the activities outlined here are self-reported and may not, in some cases, accurately reflect the activities and approaches existing in the field. It was assumed that any strategies and activities reported by NMCPs on this survey are also reflected in each country’s respective National Strategic Plan for Malaria Control, Operational Plan for Malaria Control and/or programme work plans but no analysis between the survey results and these documents was conducted. In order to improve the accuracy of the information reported, efforts were made to make sure that the respondent was the person specifically tasked with activities for populations at higher risk within the NMCP.

## Conclusions

As Asia Pacific countries adopt their national malaria elimination operational plans, it is essential they adopt methods to objectively identify higher risk populations from the current large populations stated at risk and target these groups with effective interventions. While countries have identified populations at higher risk and targeted interventions to these groups, there is limited information on the effectiveness of these interventions. Platforms like APMEN offer the opportunity for the sharing of protocols and lessons learned related to finding, targeting and successfully clearing malaria from populations at higher risk. The sharing of programme data across borders may further strengthen national and regional efforts to eliminate malaria. This exchange of real-life experience is invaluable to NMCPs when scarce scientific evidence on the topic exists to aid decision-making and can further support NMCPs to develop strategies that will deliver a malaria-free Asia Pacific by 2030.

## Abbreviations

ACT: artemisinin-based combination therapy
APMEN: Asia Pacific Malaria Elimination Network
BCC: behaviour change communication
CHW: community health worker
G6PD: glucose-6-phosphate dehydrogenase
GIS: geographic information system
GMS: greater mekong sub-region
IEC: information, education, and communication
IRS: indoor residual spraying
ITN: insecticide-treated net
LAMP: loop-mediated isothermal amplification
LLIN: long-lasting insecticide-treated net
MDA: mass drug administration
M&E: monitoring and evaluation
NMCP: National Malaria Control Programme
PCR: polymerase chain reaction
POR: prevention of reintroduction
RACD: reactive case detection
RDT: rapid diagnostic test
RBM: Roll Back Malaria Partnership
SMS: short message service
WHO: World Health Organization
